# Death from Prolonged Irritation of the Left Phrenic Nerve

**Published:** 1861-07

**Authors:** J. H. Hollister


ARTICLE II.

### DEATH FROM PROLONGED IRRITATION OF THE LEFT PHRENIC NERVE.

Reported by J. H. HOLLISTER, M. D.

The pathological symptoms in the following case were so marked, as to lead to a correct diagnosis of the difficulty, and so plainly attest the function of the phrenic nerve, that I am induced to present the facts for publication.

*Case.*—M. E., American; aged thirty-three; height six feet; figure rather slight, but finely developed; remarkably athletic.

I saw this gentleman in consultation with his father, who is a physician, on the 17th of February, 1860, and received

*The Patient's History of the Case:*—He had enjoyed uninterrupted good health until the previous July. During this month, he was twice ill, but was unable to give a clear statement of the nature of the attacks. In each instance he fainted, and was *bled.* In October, nearly three months later, while in a standing position, and exerting himself to the utmost, in raising a weight above his head, he fell senseless to the ground. His consciousness returned in a short time, and he was removed to his house and placed in bed. He suffered most excruciating pain in the region of the sixth dorsal vertebra, and was obliged to lie, for many days, nearly upon his face. His mental powers, during this period, were not impaired; there was no paralysis of the limbs, nor derangement of secreting organs. He was entirely unable to turn himself, however, from the most excruciating pain, which the least flexion of the spine produced. He was treated for local injury, and though always suffering severely from the least pressure upon the spinous process of the vertebra referred to, had so far recovered, as in January following, to make a single carriage drive of thirty miles. His business demanded his undivided attention, and while thus engaged, having had neither rest nor food since the exertion of riding, he was seized with convulsions, from which he rallied in about one hour. He soon recovered sufficiently to return home, and appeared much the same, as for the previous two months. Sixteen days later, he had a second attack, with slight tetanic spasms, and loss of consciousness for a little time. Twenty-three days from this time, he was somewhat recovered, and had accomplished a journey of two hundred miles to this city.

*Appearance.*—At the time of my first visit, he had the general symptoms of prostration which might be anticipated.

His appetite was failing, and gradual emaciation showed a failure of the assimilative powers. The peculiar symptom which first attracted my attention, was a *hacking cough* with every inspiration. There was no cessation, day nor night, and the patient was perfectly prostrated from constant effort, which almost entirely deprived him of sleep. A careful examination revealed no disease of the lungs, but the fact that every movement of the diaphragm produced a spasmodic expulsion of the inhaled air. The difficulty yielded to no treatment which we could devise. The cough was more prolonged, and excited by almost every breathing. The patient rapidly failed, literally *worn down* from want of rest. A week later the cough was less, but *now*, there was, with each descent of the diaphragm, a violent effort at vomiting. Little, if any, food or drink was retained, and exhaustion was rapid.

The vomiting changed, as the patient sunk, into a most distressing hiccough, which continued for some two days, when he expired.

*Diagnosis.*—There had been no derangement of organic functions to cause apprehension, from the time I first saw him. He evidently died from the continued spasmodic action of the diaphragm disturbing 1st, the respiration, 2d, the normal action of the stomach, and 3d, where debility was now marked—ending in simple spasmodic hiccough. The sensitiveness over the sixth dorsal vertebra had remained, though less marked than formerly. It seemed evident that by reason of injury to the spinal cord, for some reason which we could not explain, the cervical plexus of nerves must be sympathetically affected, and the irritation transmitted by one or both of the phrenic nerves to the place of distribution.

*Antopsy:*—Eighteen hours after death. Body much emaciated ; in other respects, there were no unusual appearances.

Several of the neural arches being removed in the dorsal region, the left lamina of the sixth vertebra was found fractured transversely, and without any evidence of an effort at reunion, although the injury was of nearly eight months standing!

There was a small amount of dark deposit within the arch, resulting from partial re-absorption of more or less blood-clot.

The arachnoid membrane was considerably thickened, but the cord itself presented no abnormal appearance, except a slight discoloration. We now turned the body, to trace the phrenic nerves in their course through the thorax. And here, the immediate cause of death seemed evident. Upon the left side, upon the anterior surface of the pulmonary aorta, near its base, as the nerve passed between the pleural and pericardial structures, it was raised from its ordinary course nearly half an inch, by an *enlarged lymphatic* gland. The tumor had a broad base, with a sharp ridge-like apex, dense, and unyielding, over which the nerve passed, so tightly drawn as to be imbedded about the depth of its size in the abnormal growth. The tumor, separate from other tissues, weighed *half an ounce.* The nerve presented no unusual appearance above or below the points of pressure, but when traced to its distribution, upon the upper surface of the diaphragm, there was brought to our view one of the most interesting pathological appearances I have ever beheld. We traced the nerve with care, from the point of its contact with the diaphragm, in its distribution beneath the pleural covering. And as from a centre, there was, in the direction of the larger branches of the nerve, a most beautiful capillary injection, deeply *arterial* in color, near the central point, and shading in finer distributions, till lost in the pale rose-color of the tissues around. An injection more beautiful can hardly be conceived. A faint resemblance is found in some of the finer specimens of the *Red Sea Algæ*, when skilfully displayed and dried upon paper.

No other abnormal appearances were found in any of the viscera. Here had been a train of symptoms which could be perfectly accounted for, by the appearances before us—the evidence of *nerve irritation* being *unquestionable*, and we are only left to *speculate* upon this question—Was the enlargement of the lymphatic gland a matter of accident independent of the spinal injury? or *was the nerve sympathetically affected, and this gland induced to take on abnormal growth by reason of this irritation?*

---

Dr. Thomas Turner, Passed Assistant-Surgeon in the Naval Laboratory, has seen old sailors cut potatoes into slices, and then pack them in a barrel with molasses. The molasses preserves the potatoes, and the sailors eat them raw, as an anti-scorbutic.—*Hamilton's Military Surgery.*

